# Highly Pathogenic Avian Influenza A(H5N8) Virus in Swans, China, 2020

**DOI:** 10.3201/eid2706.204727

**Published:** 2021-06

**Authors:** Xiang Li, Xinru Lv, Yi Li, Peng Peng, Ruifang Zhou, Siyuan Qin, Enda Ma, Wenqiang Liu, Tian Fu, Peiran Ma, Qing An, Yiran Li, Yuping Hua, Yulong Wang, Chengliang Lei, Dong Chu, Heting Sun, Yanbing Li, Yuwei Gao, Hongliang Chai

**Affiliations:** Northeast Forestry University College of Wildlife and Protected Area, Harbin, China (X. Li, X. Lv, Y. Li, T. Fu, P. Ma, Q. An, Yiran Li, Y. Hua, Y. Wang, H. Chai);; General Station for Surveillance of Wildlife Disease and Wildlife Borne Diseases, National Forestry and Grassland Administration, Shenyang, China (P. Peng, S. Qin, D. Chu, H. Sun);; Bayannur Desert Comprehensive Management Center, Bayannur Forestry Scientific Research Institute, Bayannur, China (R. Zhou);; Wildlife and Wetland Conservation Center, Bayannur Forestry and Grassland Administration, Bayannur (E. Ma, W. Liu);; State Key Laboratory of Veterinary Biotechnology, Harbin Veterinary Research Institute, Harbin (Yanbing Li);; National Forestry and Grassland Administration Department of Wildlife Protection, Beijing, China (C. Lei);; Military Veterinary Research Institute of Academy of Military Medical Sciences, Changchun, China (Y. Gao)

**Keywords:** influenza A virus, H5N8 subtype, avian influenza, Phylogenyviruses, respiratory infections, zoonoses, viruses, vaccine-preventable diseases, Inner Mongolia, China, swans

## Abstract

In October 2020, highly pathogenic avian influenza A(H5N8) viruses were detected in 2 dead swans in Inner Mongolia, China. Genetic analysis showed that the H5N8 isolates belong to clade 2.3.4.4b and that the isolates cluster with the H5N8 viruses isolated in Eurasia in the fall of 2020.

Since 2008, highly pathogenic avian influenza (HPAI) H5 clade 2.3.4 viruses with various neuraminidase combinations have been identified, and these subtypes have subsequently evolved into different subclades, including clade 2.3.4.4 (*1*–*3*). In contrast to the 2014–2015 H5N8 clade 2.3.4.4 viruses, which spread worldwide through wild migratory birds (*4*), the intercontinental spread of clade 2.3.4.4b viruses began in fall 2016 (*5*). Moreover, clade 2.3.4.4b viruses have had a sustained prevalence in Europe, Africa, and the Middle East in recent years (https://www.oie.int/en/animal-health-in-the-world). In January 2020, clade 2.3.4.4h of HPAI A(H5N6) virus was detected in whooper swans (*Cygnus cygnus*) and mute swans (*C. olor*) in Xinjiang (*6*); however, no outbreaks of H5N8 in mainland China have been reported since 2017. We report the reemergence of HPAI H5N8 viruses from wild aquatic birds in mainland China.

On October 17, 2020, we collected multiple organs (brains, larynx, liver, lung, pancreas, kidney, spleen, and rectum) from 2 dead swans, a whooper swan and a mute swan, in Wuliangsuhai Lake in Bayannur City, Inner Mongolia, China (40.95138889°N, 108.9266667°E) (Figure). We sequenced the H5N8 genomes directly from organs. We isolated 2 H5N8 influenza viruses, A/whooper swan/Inner Mongolia/W1-1/2020(H5N8) and A/mute swan/Inner Mongolia/W2-1/2020(H5N8).

**Figure:**
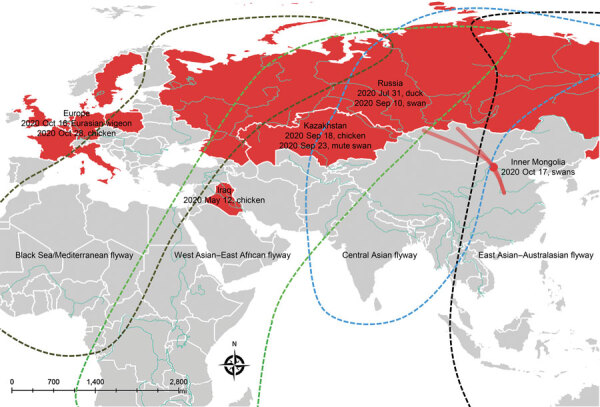
Global distribution of the influenza A(H5N8) viruses related to 2 H5N8 isolates detected in whooper swans (*Cygnus cygnus*) and mute swans (*C. olor*) in Inner Mongolia, China, 2020. Red dot indicates sampling site in Inner Mongolia; red solid lines indicate whooper swan migratory routes in central China. Dates refer to the day of initial H5N8 virus isolated in poultry and wild birds in each country in 2020.

We sequenced the full-length genomes and confirmed that the 2 isolates were HPAI viruses on the basis of the amino acid sequence REKRRKR↓GLFGAI at the hemagglutinin cleavage site. The sequences of these 2 Inner Mongolia H5N8 isolates (IM-H5N8) were deposited into the GISAID database (http://www.gisaid.org; accession nos. EPI1811641–56). The receptor-binding site at the 222–224 (H5 numbering) motif (QRG) suggested avian-like (α 2–3-SA) receptors, and the substitutions D94N, S123P, and S133A (H5 numbering), which are associated with increased binding to human-like (α 2–6-SA) receptors, were identified. The residues Q591, E627, and D701 in the polymerase basic 2 protein suggest that these viruses have not yet adapted to mammalian hosts.

Sequence comparisons showed high nucleotide identity across all 8 gene segments between the 2 IM-H5N8 isolates (99.8%–100%). A BLAST search (https://blast.ncbi.nlm.nih.gov/Blast.cgi) performed in the GISAID database suggested that IM-H5N8 shares the highest nucleotide identity (>99%) with the H5N8 viruses isolated in Eurasia in fall 2020 (EA-H5N8) (Appendix). These results indicate that IM-H5N8 and EA-H5N8 are descendants of a common ancestral virus. Phylogenetic analysis further confirmed that the 8 segments had a common evolutionary source, clustering with EA-H5N8 and belonging to clade 2.3.4.4b (Appendix).

Clade 2.3.4.4b H5N8 viruses were detected mainly in poultry in Europe in early 2020 but were not related to IM-H5N8 (Appendix) (*7*). On May 12 2020, IM-H5N8 like virus was first detected in poultry in Iraq (*7*). It might be the source of IM-H5N8–like viruses. Soon after, similar viruses were reported in southern central Russia in late July 2020, and they jumped into wild birds in September in Russia and Kazakhstan (Appendix Table 1). Migratory birds moving within several flyways in Eurasia have overlapping breeding areas (*8*), and breeding origin assignments suggest that migration distances vary by a maximum of ≈3,500 km (*9*). Such a unique ecosystem could be implicated as a pathway for the cross-regional spread of HPAI viruses during the autumn migration of waterfowl. Birds breeding in Russia that migrate along different routes might be responsible for the widespread transmission of H5N8 viruses in both Europe and China in fall 2020.

The migration routes of whooper swans along the Central Asian flyway have been identified (*10*); the widely distributed lakes in western and northern Mongolia (including the lakes in Russia near Mongolia) are important breeding areas, and Inner Mongolia (including Bayannur City, Baotou City, and Ordos City) and Shaanxi (including Yulin City) are the key stopover sites during the migration of whooper swans in China. Whooper swans usually winter along the Yellow River (e.g., in Henan [Sanmenxia Reservoir area] and Shanxi [Shengtian Lake]), and whooper swans also wander between different wintering grounds. Despite no reported outbreak of HPAI A(H5N8) virus in Mongolia, Russia reported a similar swan outbreak of HPAI A(H5N8) virus on August 28, 2020 (https://www.oie.int/wahis_2/temp/reports/en_fup_0000035737_20200915_110259.pdf), which could be the source of the infections in these swans in Bayannur City in Inner Mongolia through swan migration. 

In conclusion, we detected HPAI A(H5N8) during the autumn migration of whooper swans through Inner Mongolia. This finding warrants strengthening the monitoring of HPAI A(H5N8) in swans and other migratory waterfowl in the main stopover sites and wintering grounds, especially in the Sanmenxia Reservoir area in Henan Province.

Appendix Additional information about highly pathogenic avian influenza A(H5N8) virus in swans, China, 2020.
