# Supplementary material for: Highly Pathogenic Avian Influenza A(H5N8) Virus in Swans, China, 2020
Source: Emerg Infect Dis. 2021 Jun;27(6):1732–4. doi: 10.3201/eid2706.204727 (PMC8153893; doi:10.3201/eid2706.204727)

Article DOI: https://doi.org/10.3201/eid2706.204727

Highly Pathogenic Avian Influenza A (H5N8) Virus in Swans, China, 2020

Appendix

Materials and Methods

Samples

Two sick swans, whooper swan (*Cygnus cygnus*) and mute swan (*Cygnus olor*) were found almost at the same site in Wuliangsuhai Lake in Bayannur city, Inner Mongolia, China (41.826234°N, 107.54972°E) on 17 October 2020. The swans died soon and virus was detected and collected from organs at the same day. We collected multiple organs (brains, larynx, liver, lung, pancreas, kidney, spleen and rectum) from two dead swans. We sequenced the H5N8 genomes directly from organs, and we used them in whole genetic analysis. The egg passages were used to confirm the genome and to get the long-term preserved viral strains. We inoculated 10-day-old specific pathogen-free chicken embryos (National Poultry Laboratory Animal Resource Center, Harbin Veterinary Research Institute, Chinese Academy of Agriculture Sciences, Harbin 150069, China) with the homogenates of mixed organs, respectively. All chicken embryos dead within 48 hours, the allantoic fluid was harvested, and the hemagglutinin (HA) activity was assayed. Subtypes of influenza viruses were identified initially by using the hemagglutination inhibition (HI) test. Viral RNA was extracted from organs or HA positive samples from incubated allantoic fluid using a QIAamp Viral RNA Mini Kit (Qiagen, Germany), reverse transcribed using the primer Un12 and subjected to RT-PCR using the method described in the WHO manual (World Health Organization [WHO], 2002) to further confirm AIV positive. The PCR products of eight fragments of the isolates were sequenced using a set of specific sequencing primers listed in a previous dissertation (*1*). The sequence data were compiled using the SeqMan program (DNASTAR, Madison, WI, United States). Two H5N8 influenza viruses, A/whooper swan/Inner Mongolia/W1–1/2020(H5N8) and A/mute swan/Inner Mongolia/W2–1/2020(H5N8), were isolated.

Genetic Analysis

A BLASTn search was performed against sequences in the GISAID database to identify the closest relatives of the two Inner Mongolia H5N8 isolates (QH-H5N8) in early January 2021, and eight datasets were derived from the top 100 BLASTn hits. The genome of clade 2.3.4.4 H5N8 viruses isolated in 2020 were also downloaded. Sequences were aligned using MAFFT (*2*) implemented in PhyloSuite 1.21 (*3*). The alignment lengths for each dataset were: PB2 2,277 nt (nt), PB1 2,271 nt, PA 2,148 nt, HA 1,681 nt, NP 1,494 nt, NA 1,407 nt, M 979 nt, NS 835 nt. For all eight datasets, sequences without full alignment length were removed. We reconstructed the phylogenetic trees using selected representative sequences of 2.3.4.4 and sequences derived from GISAID. Maximum likelihood phylogenies were inferred using IQ-TREE (*4*) under the best-fit substitution model for 10000 ultrafast bootstraps (*5*). Best-fit substitution model was selected using the Bayesian information criterion by ModelFinder (*6*) implemented in PhyloSuite 1.21 (*3*).

References

1. Chai H. Molecular epidemiological study on influenza virus in wild birds of Heilongjiang. Harbin, China: Northeast Forestry University; 2012.

2. Katoh K, Standley DM. MAFFT multiple sequence alignment software version 7: improvements in performance and usability. Mol Biol Evol. 2013;30:772–80. PubMed https://doi.org/10.1093/molbev/mst010

3. Zhang D, Gao F, Jakovlić I, Zou H, Zhang J, Li WX, et al. PhyloSuite: An integrated and scalable desktop platform for streamlined molecular sequence data management and evolutionary phylogenetics studies. Mol Ecol Resour. 2020;20:348–55. PubMed https://doi.org/10.1111/1755-0998.13096

4. Nguyen LT, Schmidt HA, von Haeseler A, Minh BQ. IQ-TREE: a fast and effective stochastic algorithm for estimating maximum-likelihood phylogenies. Mol Biol Evol. 2015;32:268–74. PubMed https://doi.org/10.1093/molbev/msu300

5. Minh BQ, Nguyen MA, von Haeseler A. Ultrafast approximation for phylogenetic bootstrap. Mol Biol Evol. 2013;30:1188–95. PubMed https://doi.org/10.1093/molbev/mst024

6. Kalyaanamoorthy S, Minh BQ, Wong TKF, von Haeseler A, Jermiin LS. ModelFinder: fast model selection for accurate phylogenetic estimates. Nat Methods. 2017;14:587–9. PubMed https://doi.org/10.1038/nmeth.4285

Appendix Table 1. Virus isolates sharing the highest nucleotide similarity (top 2) with the two Inner Mongolia H5N8 isolates as identified on global initiative on sharing all influenza data (GISAID) in early January 2021

Segment	Virus name	Accession number*	similarity
A/whooper swan/Inner Mongolia/W1–1/2020(H5N8) [(A/mute swan/Inner Mongolia/W2–1/2020(H5N8)]			
PB2	A/Greylag goose/England/033100/2020(H5N8)	EPI1837929	99%
	A/barnacle goose/Sweden/SVA201117SZ0468/KN003355/2020(H5N8)	EPI1814739	99%
PB1	A/goose/Russia Novosibirsk region/1–12/2020(H5N8)	EPI1839239	99%
	A/domestic duck/Kazakhstan/1–274–20-B/2020(H5N8)	EPI1811615	99%
PA	A/domestic duck/Kazakhstan/1–274–20-B/2020(H5N8)	EPI1811615	99%
	A/goose/Russia Novosibirsk region/1–12/2020(H5N8)	EPI1839239	99%
HA	A/goose/Russia Novosibirsk region/1–12/2020(H5N8)	EPI1839239	99%
	A/domestic duck/Kazakhstan/1–274–20-B/2020(H5N8)	EPI1811615	99%
NP	A/duck/Russian Federation Omsk/1328–2/2020(H5N8)	EPI1811690	99%
	A/domestic duck/Kazakhstan/1–274–20-B/2020(H5N8)	EPI1811615	99%
NA	A/turkey/England/038115/2020(H5N8)	EPI1837953	99%
	A/turkey/England/037784/2020(H5N8)	EPI1837937	99%
M	A/barnacle goose/Sweden/SVA201117SZ0468/KN003355/2020(H5N8)	EPI1814739	99%
	A/Greylag_goose/England/033100/2020(H5N8)	EPI1837929	99%
NS	A/chicken/England/037052/2020(H5N8)	EPI1837911	99%
	A/Eurasian wigeon/Italy/20VIR7301–34/2020(H5N8)	EPI1815377	99%

*EpiFlu Database of Global Initiative on Sharing All Influenza Data (GISAID).

Appendix Table 2. Information of viruses related the two Inner Mongolia isolates according to the HA genes

Date	Location	Isolate name	Isolate ID
2020-May-12	Iraq	A/chicken/Iraq/1/2020(H5N8)	EPI_ISL_623074
2020-Sep-18	Kazakhstan	A/chicken/Kazakhstan/Kn-3/2020(H5N8)	EPI_ISL_739686
2020-Sep-18	Kazakhstan	A/chicken/Kazakhstan/Kn-6/2020(H5N8)	EPI_ISL_739687
2020-Sep-19	Kazakhstan	A/domestic goose/Kazakhstan/1–242_2–20-B/2020(H5N8)	EPI_ISL_615073
2020-Sep-20	Kazakhstan	A/domestic goose/Kazakhstan/1–248_2–20-B/2020(H5N8)	EPI_ISL_615068
2020-Sep-22	Kazakhstan	A/domestic chicken/Kazakhstan/1–261_1–20-B/2020(H5N8)	EPI_ISL_615070
2020-Sep-23	Kazakhstan	A/mute swan/Kazakhstan/1–267–20-B/2020(H5N8)	EPI_ISL_614401
2020-Sep-25	Kazakhstan	A/domestic duck/Kazakhstan/1–274–20-B/2020(H5N8)	EPI_ISL_615072
2020-Jul-31	Russia	A/duck/Chelyabinsk/1207–1/2020(H5N8)	EPI_ISL_637098
2020-Aug-17	Russia	A/duck/Russian Federation Omsk/1328–2/2020(H5N8)	EPI_ISL_626650
2020-Sep-10	Russia	A/swan/Tumen/1479–2/2020(H5N8)	EPI_ISL_661178
2020-Sep-15	Russia	A/goose/Russia Novosibirsk region/1–12/2020(H5N8)	EPI_ISL_739684
2020-Sep-18	Russia	A/duck/Russian Federation/Saratov/1578–2/2020 (H5N8)	EPI_ISL_626649
2020-Sep-20	Russia	A/chicken/Russia Novosibirsk region/3–1/2020(H5N8)	EPI_ISL_739690
2020-Sep-20	Russia	A/chicken/Russia Novosibirsk region/3–15/2020(H5N8)	EPI_ISL_739691
2020-Sep-20	Russia	A/chicken/Russia Novosibirsk region/3–29/2020(H5N8)	EPI_ISL_739692
2020-Sep-22	Russia	A/chicken/Russia Novosibirsk region/1910–1/2020(H5N8)	EPI_ISL_739688
2020-Sep-22	Russia	A/chicken/Russia Novosibirsk region/1910–2/2020(H5N8)	EPI_ISL_739689
2020-Oct-02	Russia	A/chicken/Russian Federation/Omsk/1680–10/2020(H5N5)	EPI_ISL_626647
2020-Oct-02	Russia	A/goose/Russian Federation/Omsk/1680–6/2020(H5N5)	EPI_ISL_626648
2020-Oct-16	Netherlands	A/Eurasian Wigeon/Netherlands/1/2020(H5N1)	EPI_ISL_603133
2020-Oct-16	Netherlands	A/Eurasian Wigeon/Netherlands/4/2020(H5N1)	EPI_ISL_603134
2020-Oct-16	Netherlands	A/Eurasian Wigeon/Netherlands/5/2020(H5N1)	EPI_ISL_603135
2020-Oct-16	Netherlands	A/Eurasian Wigeon/Netherlands/7/2020(H5N8)	EPI_ISL_603136
2020-Oct-17	Netherlands	A/mute swan/Netherlands/20015931–001/2020(H5N8)	EPI_ISL_591075
2020-Oct-28	Netherlands	A/chicken/Netherlands/20016597–026030/2020(H5N8)	EPI_ISL_603132
2020-Oct-28	Netherlands	A/greylag goose/Netherlands/20016582–004/2020(H5N1)	EPI_ISL_632314
2020-Nov-01	Netherlands	A/barnacle goose/Netherlands/20016935–002/2020(H5N8)	EPI_ISL_632317
2020-Nov-01	Netherlands	A/greylag goose/Netherlands/20016879–001/2020(H5N8)	EPI_ISL_632318
2020-Nov-02	Netherlands	A/eurasian teal/Netherlands/20016896–013/2020(H5N1)	EPI_ISL_632315
2020-Nov-02	Netherlands	A/chicken/Netherlands/20016978–001/2020(H5N8)	EPI_ISL_641377
2020-Nov-10	Netherlands	A/chicken/Netherlands/20017639–001/2020(H5N8)	EPI_ISL_641394
2020-Nov-11	Netherlands	A/chicken/Netherlands/20017694–004/2020(H5N8)	EPI_ISL_641395
2020-Dec-14	Netherlands	A/chicken/Netherlands/20019879–001005/2020(H5N1)	EPI_ISL_711055
2020-Oct-29	Germany	A/buzzard/Germany-MV/AI02166/2020(H5N5)	EPI_ISL_614399
2020-Oct-30	Germany	A/barnacle goose/Germany-SH/AI02167/2020(H5N8)	EPI_ISL_614400
2020-Oct-30	Denmark	A/peregrine falcon/Denmark/13776–1/2020–10–30(H5N5)	EPI_ISL_644737
2020-Nov-04	Denmark	A/barnacle goose/Denmark/14138–1/2020–11–04(H5N8)	EPI_ISL_644824
2020-Oct-30	England	A/Greylag goose/England/033100/2020(H5N8)	EPI_ISL_710508
2020-Nov-02	England	A/chicken/England/030720/2020(H5N8)	EPI_ISL_626652
2020-Nov-03	England	A/Canada goose/England/032697/2020(H5N8)	EPI_ISL_710506
2020-Nov-03	England	A/Greylag goose/England/032698/2020(H5N8)	EPI_ISL_710507
2020-Nov-09	England	A/chicken/England/033708/2020(H5N8)	EPI_ISL_710509
2020-Nov-18	England	A/whistling duck/England/035643/2020(H5N8)	EPI_ISL_710512
2020-Nov-19	England	A/chicken/England/037052/2020(H5N8)	EPI_ISL_710511
2020-Nov-24	England	A/mute swan/Wales/048068/2020(H5N5)	EPI_ISL_683999
2020-Nov-28	England	A/turkey/England/037784/2020(H5N8)	EPI_ISL_710504
2020-Dec-02	England	A/turkey/England/038115/2020(H5N8)	EPI_ISL_710505
2020-Nov-04	Belgium	A/Cygnus olor/Belgium/11956_001/2020(H5N8)	EPI_ISL_644735
2020-Nov-07	Belgium	A/Numenius arquata/Belgium/11956_003/2020(H5N8)	EPI_ISL_664102
2020-Nov-07	Belgium	A/Anser albifrons/Belgium/11956_005/2020(H5N8)	EPI_ISL_661313
2020-Nov-18	Belgium	A/Gallus gallus/Belgium/12168_002/2020(H5N5)	EPI_ISL_660264
2020-Nov-10	Sweden	A/Peregrine falcon/Sweden/SVA201117SZ0467/20KN003345/2020(H5N8)	EPI_ISL_668456
2020-Nov-12	Sweden	A/barnacle goose/Sweden/SVA201117SZ0468/KN003355/2020(H5N8)	EPI_ISL_668457
2020-Nov-13	Sweden	A/Turkey/Sweden/SVA201114SZ0001/20KN303106/2020(H5N8)	EPI_ISL_647969
2020-Nov-10	France	A/chicken/France/20P016448/2020(H5N8)	EPI_ISL_667810
2020-Nov-14	Italy	A/mallard/Italy/20VIR7139–124_feather/2020(H5N8)	EPI_ISL_683594
2020-Nov-14	Italy	A/mallard/Italy/20VIR7139–73/2020(H5N8)	EPI_ISL_654958
2020-Nov-14	Italy	A/Eurasian wigeon/Italy/20VIR7139–121/2020(H5N8)	EPI_ISL_683593
2020-Nov-21	Italy	A/Eurasian wigeon/Italy/20VIR7301–206/2020(H5N1)	EPI_ISL_683592
2020-Nov-21	Italy	A/Eurasian wigeon/Italy/20VIR7301–31/2020(H5N8)	EPI_ISL_683751
2020-Nov-21	Italy	A/Eurasian wigeon/Italy/20VIR7301–34/2020(H5N8)	EPI_ISL_683752
2020-Nov-24	Poland	A/chicken/Poland/448/2020(H5N8)	EPI_ISL_661177
2020-Dec-01	Poland	A/turkey/Poland/464/2020(H5N8)	EPI_ISL_779129

Appendix Figure. Maximum-likelihood phylogenetic trees. Our Inner Mongolia H5N8 isolates were marked with red circle and viruses isolated in 2020 are shown in blue. A UFBoot support values ≥90 was shown. Segments shown: A) polymerase basic; B) polymerase basic; C) polymerase; D) hemagglutinin; E) nucleoprotein; F) neuraminidase; G) matrix protein; H) nonstructural protein.

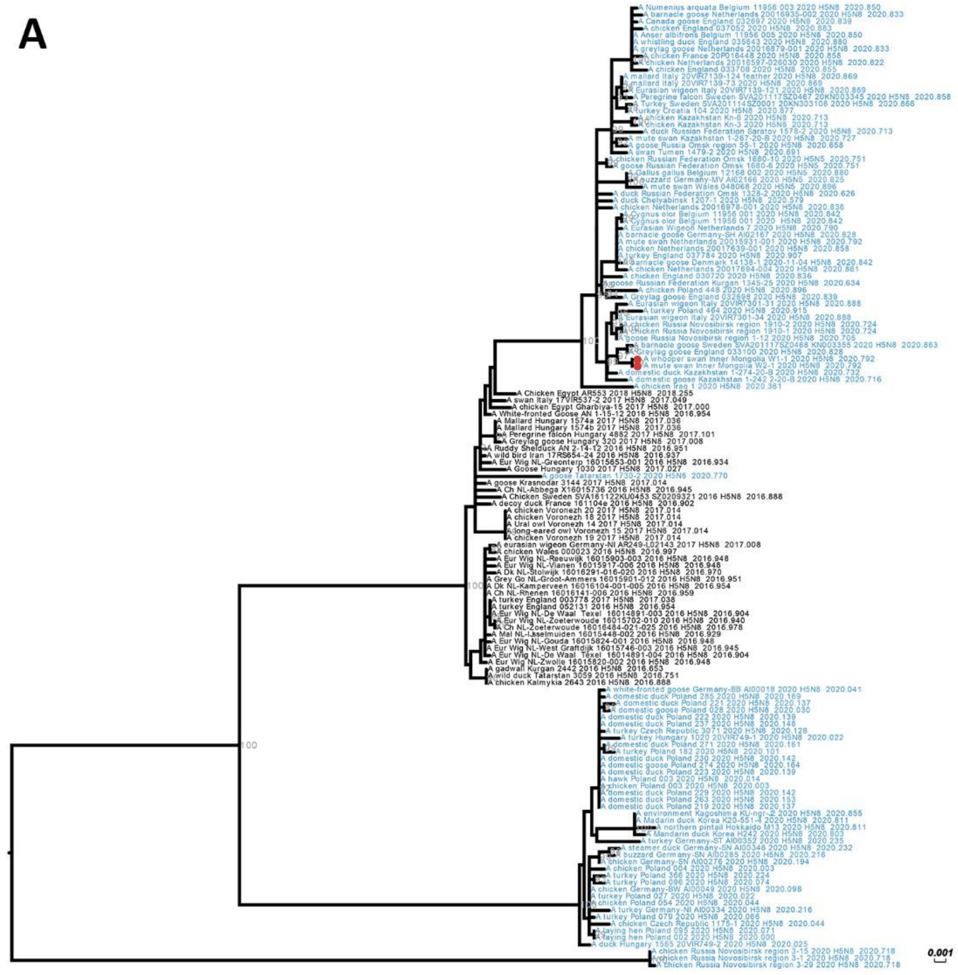

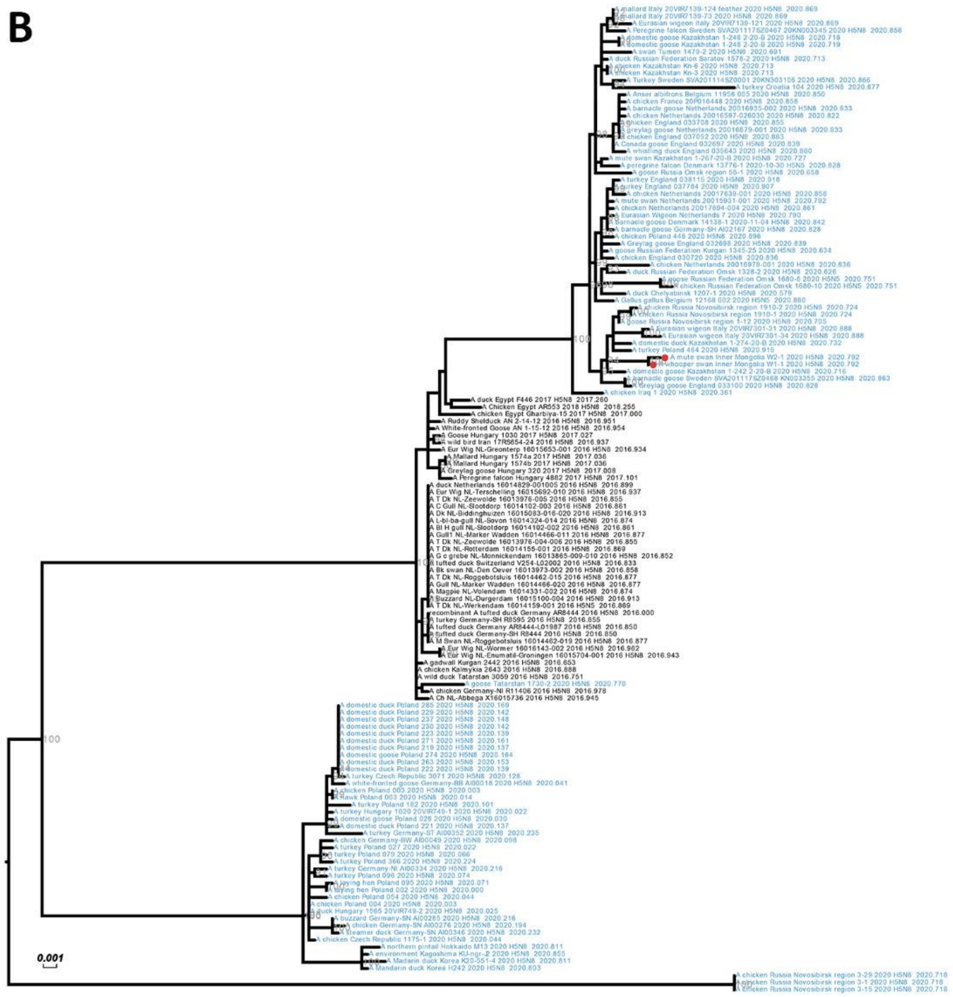
B
0.001

C

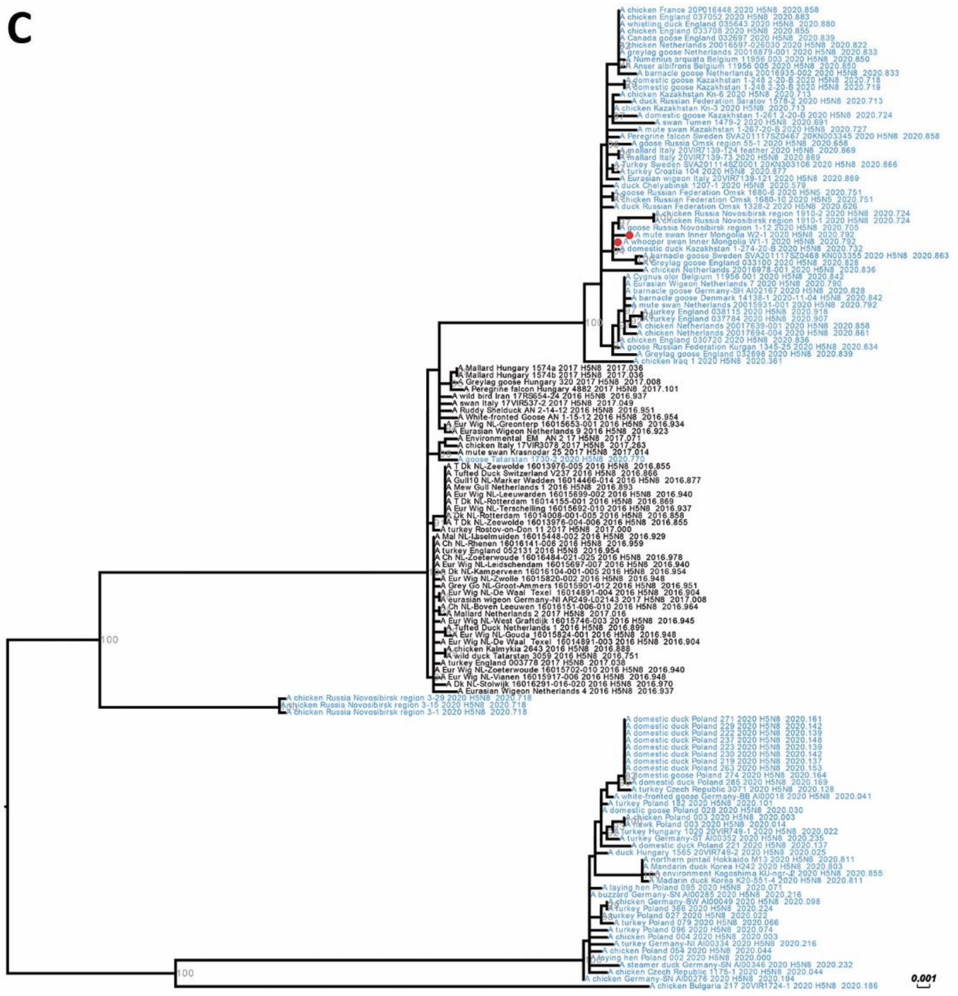

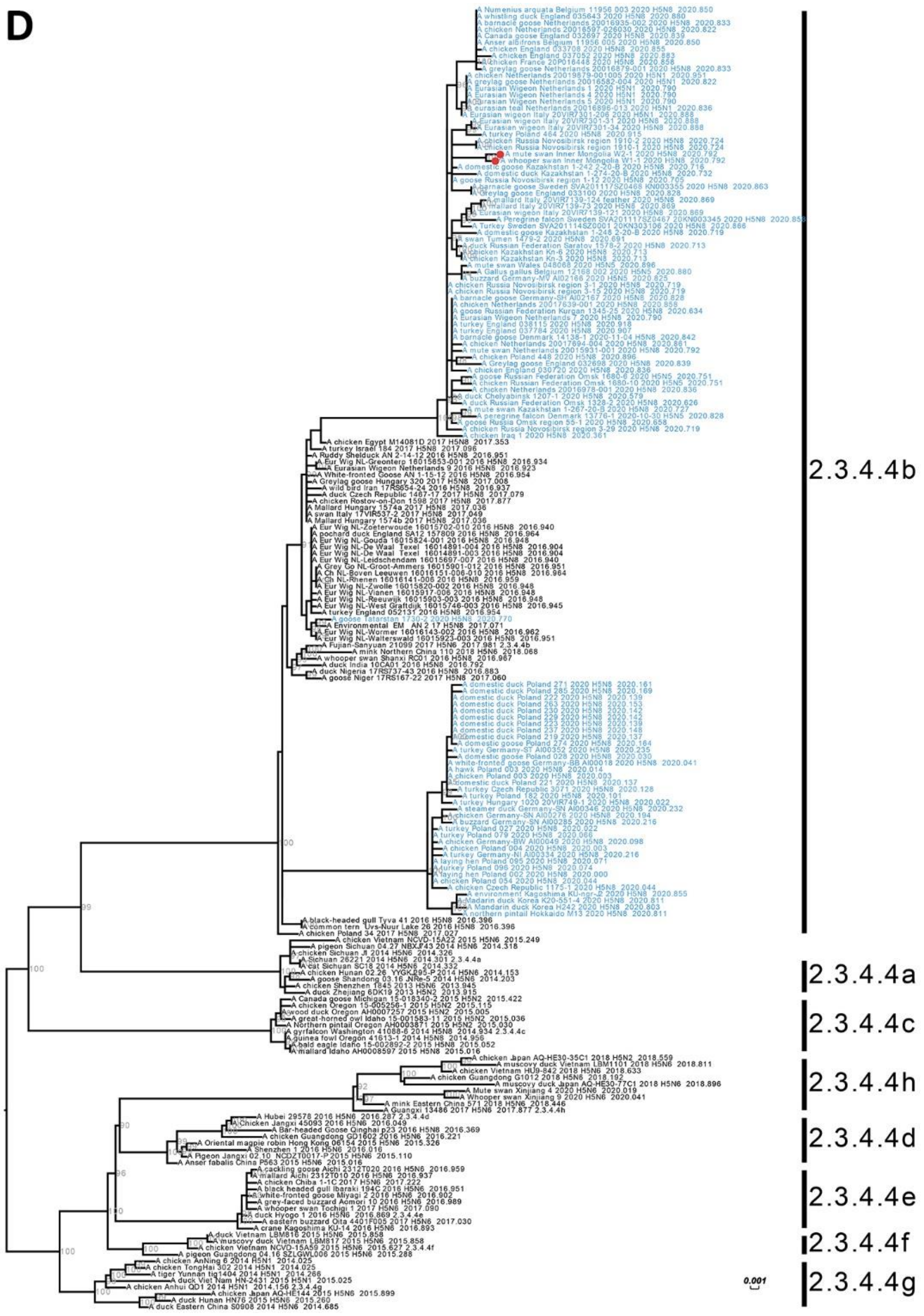
D
2.3.4.4b
2.3.4.4a
2.3.4.4c
2.3.4.4h
2.3.4.4d
2.3.4.4e
2.3.4.4f
2.3.4.4g
0.001

E

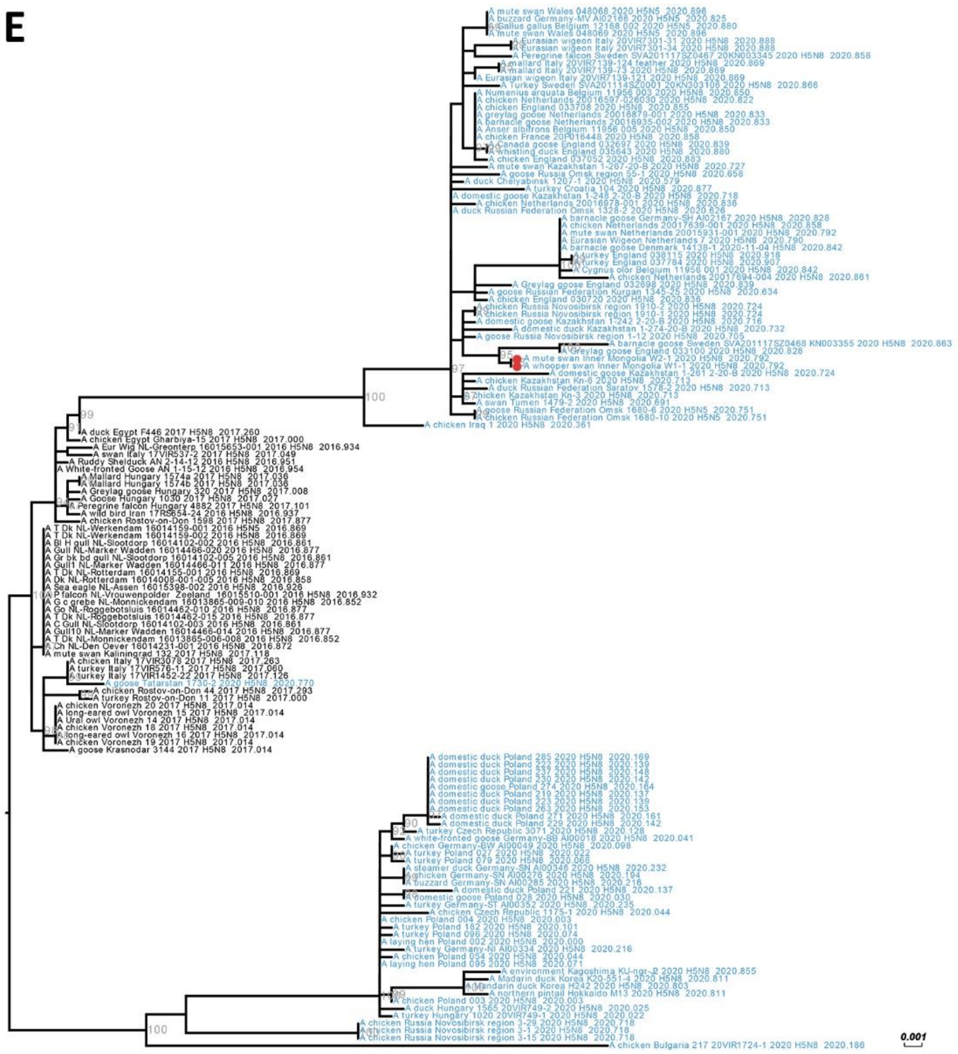

F

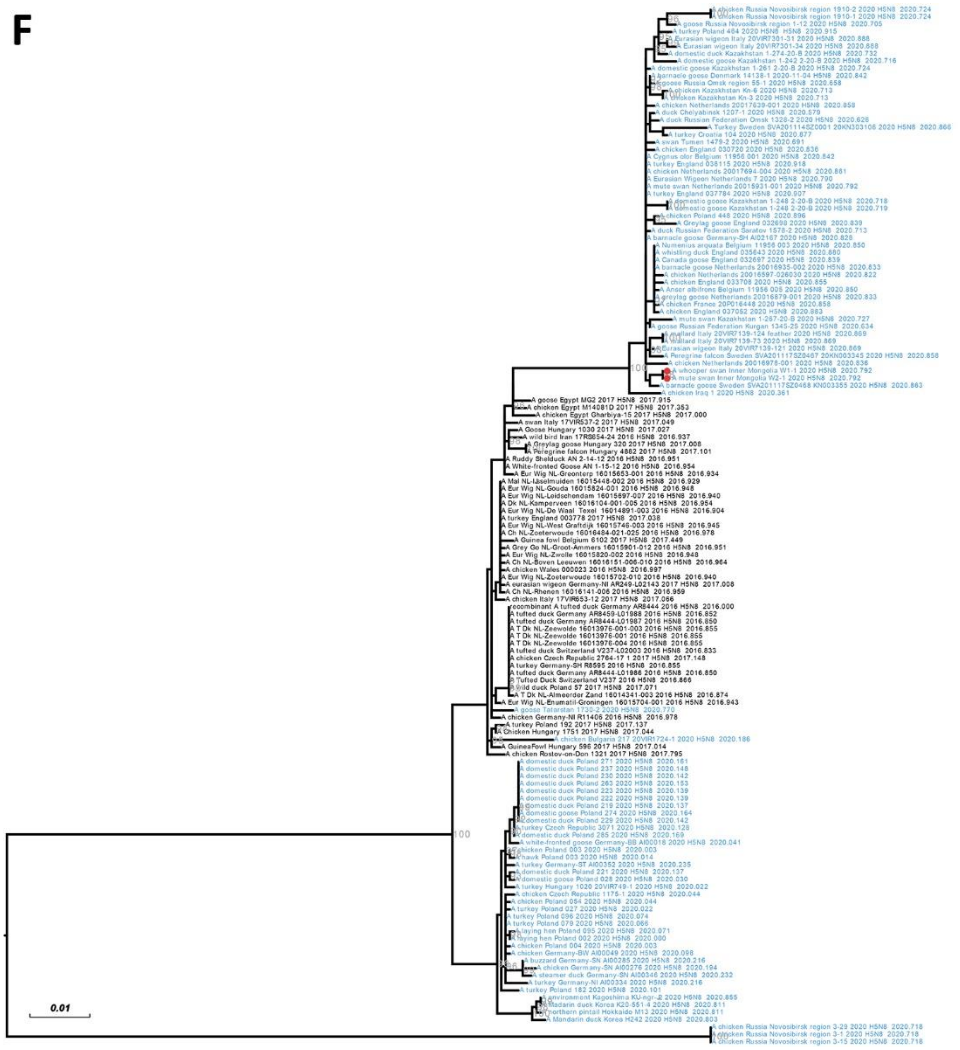

G

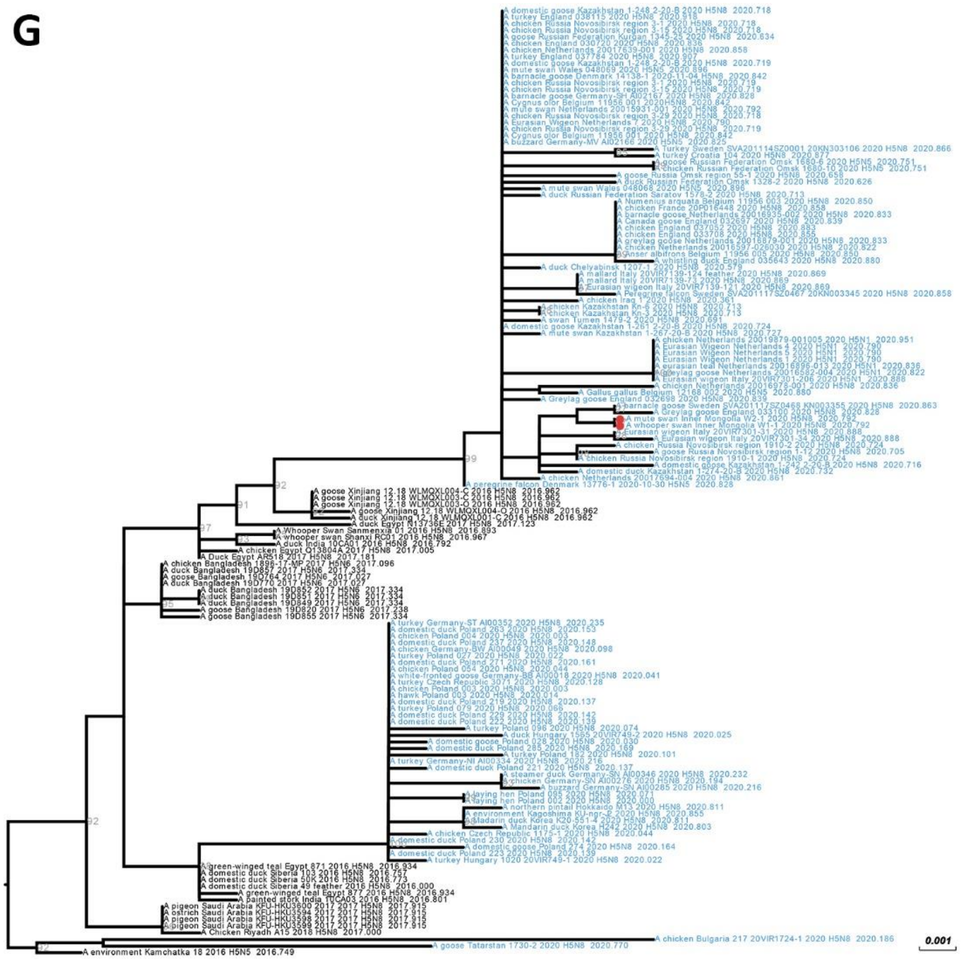

H

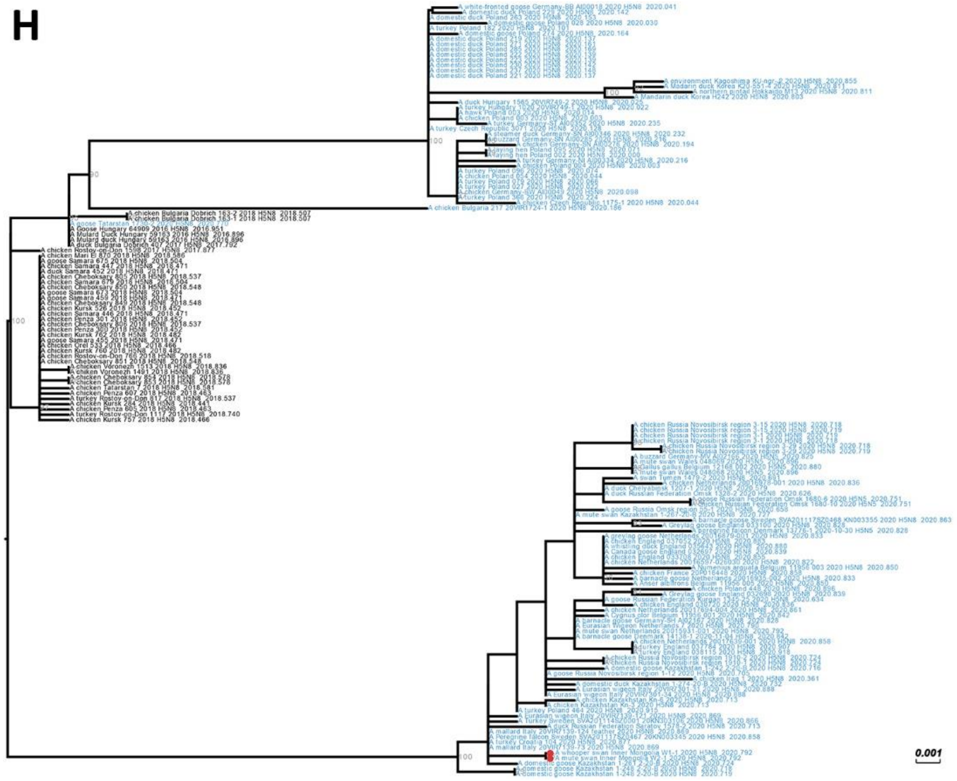

Supplement: Appendix — Additional information about highly pathogenic avian influenza A(H5N8) virus in swans, China, 2020. [file 20-4727-Techapp-s1.pdf]
